# Doppler findings in a rare Coronary Artery Fistula

**DOI:** 10.1186/1476-7120-5-10

**Published:** 2007-03-09

**Authors:** Christian Jung, Carl Jorns, James Huhta

**Affiliations:** 1 Pediatric Cardiology, All Children's Hospital, University of South Florida, 801 6^th ^Street South, St. Petersburg, FL 33701, USA; 2 Department of Internal Medicine, I Division of Cardiology, University of Jena, Erlanger Allee 101, 07747 Jena, Germany

## Abstract

One of the primary forms of congenital anomalies of the coronary arteries is coronary artery fistula (CAF). It is defined as a direct communication between the coronary artery and any surrounding cardiac chamber or vascular structure, which bypasses the myocardial capillary bed. We present a newborn baby with a large coronary artery fistula connecting the left anterior descending (LAD) artery to the left ventricular (LV) apex. Associated cardiac abnormalities were found: a ventricular septal defect (diameter 4 mm), a patent foramen ovale as well as trivial tricuspid and mitral regurgitation. Here we demonstrate the echocardiograms of an extremely rare form of CAF diagnosed within the first days of postnatal life.

## Background

Normal coronary artery anatomy is characterized by two ostia placed centrally in the right and left sinus of Valsalva. The three main coronary arteries branch superiorly to the atria and inferiorly to the ventricles; they end in broom-like arborizations, which penetrate the myocardium [1]. Clinical suspicion that a patient's problem may be the result of coronary anomalies remains an important challenge in diagnosis, especially in children. Hemodynamically significant congenital anomalies of coronary arteries are shown in Table 1[2]. One of the primary forms is coronary artery fistula (CAF), that are defined as a direct communication of a coronary artery with a cardiac chamber, great vessel or other vascular structure, bypassing the myocardial capillary bed. Embryologically, these fistulae seem to represent persistent junctions of primordial epicardial vessels with intramyocardial sinusoidal circulation. CAF accounts for 0.27–0.40% of all congenital defects [3,4] and was first described by Krause in 1865 [5]. Some studies showed that the source is most often the right coronary artery (RCA) with the left coronary artery (LCA) being much less involved [6], while other studies found that the origin is 50% the RCA and 50% LCA [7-9]. Furthermore, there are studies showing that the LCA is more often the origin than RCA [10]. The exit of the CAF is in decreasing order of frequency the right ventricle, right atrium and the pulmonary artery [7,8,11]. Consistent in all studies is that the LV is a very uncommon exit of a CAF [7-11]. Considering the rarity of this, we present a case of a CAF with the diagnosis and echocardiogradphic evaluation within the first days of life.

**Table 1:** Hemodynamically significant congenital anomalies of the coronary arteries

**Isolated/primary – without CHD:**	**Secondary – with CHD:**
• Congenital coronary artery fistula	• PA + IVS
• Anomalous origin of accessory coronary arteries from the pulmonary artery	• AA + MS
• Ectopic origin of the coronary arteries from aortic sinus	
• Absence of a coronary artery	

AA: aortic atresia; CHD: congenital heart disease; IVS: interventricular septum; MS: mitral stenosis; PA: pulmonary atresia – modified from [2]

## Case Report

**Figure 1:**
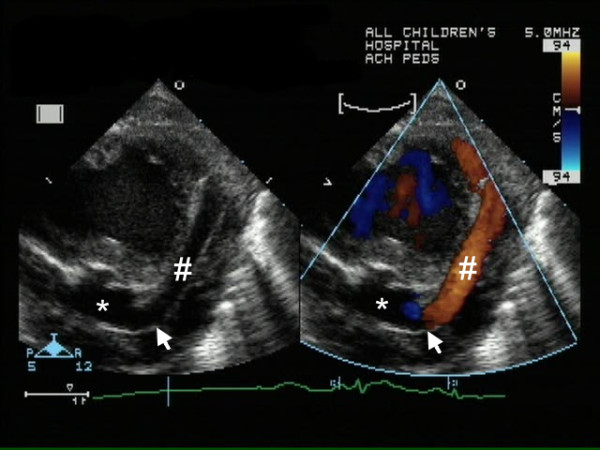
Two dimensional (left) and color Doppler (right) on day one (atypical short axis view of LV). The large coronary artery fistula drains (arrow) into the left ventricular apex (*). The LAD (#) was dilated up to 4.4 mm in diameter along its course.

We report about a newborn baby which was noted to have a murmur on the first day of life. The child was 39 weeks of gestation and was born by repeat cesarean section to a 26-year-old gravida 4, para 3 mother. Apgar scores were 9 and 9, the birth weight was 3.57 kg. The family history was significant for a sister with a PDA, status post ligation. The physical examination was negative except for a 3/6 holosystolic murmur at the mid left sternal border. An echocardiogram (performed with HP Sonos 7500, 5 MHz transducer, Philips Medical Systems, Germany) demonstrated a large coronary artery fistula connecting the left anterior descending artery to the left ventricular apex. The proximal coronary artery measured 4.4 mm in diameter and was dilated along its course (Figure 1) and showed a diastolic flow in the Doppler evaluation. A moderate-sized apical muscular ventricular septal defect (VSD; diameter 4 mm) was seen. There was also a patent foramen ovale (PFO) as well as trivial tricuspid and mitral regurgitation. The right ventricular pressures appeared elevated and were estimated to be near systemic. No evidence of regional wall motion abnormalities suggestive of ischemia was seen. Due to the risk for ischemia secondary to steal from the coronary fistula, daily electrocardiograms were obtained after admission to the cardiovascular intensive care unit (CVICU) for monitoring. At no point of time during the patient's care a pathological ECG was obtained. Due to the infant demonstrating mild and intermittent tachypnea, diuretic therapy was started on day five. The child was discharged home on day 8 without any complications on diuretic therapy and monitored closely on an ambulant manner as an attempt for conservative therapy on request of the parents.

## Discussion

CAF diagnosed in the neonate is very rare. Symptoms of the disease at older age may be continuous systolic-diastolic murmur, congestive heart failure, pulmonary hypertension, bacterial endocarditis, premature arteriosclerotic changes within the fistulae and thromboembolic events. Angina is uncommon and myocardial infarction rare. It is postulated that these ischaemic symptoms are caused by coronary steal [12]. If the fistula drains into a right-side chamber or vessel, including the right atrium, right ventricle, or pulmonary artery, it can cause a left to right shunt with increased flow to the pulmonary circulation and ultimately to the left heart. If the fistula drains into the left atrium and ventricle, it can produce an isolated volume overload of those chambers similar to the overload that occurs in aortic reurgitation [13]. A CAF that has not been detected or closed in childhood has been reported to become symptomatic in adulthood because of this chronic volume load and ischaemia. Therefore it has been recommended that these fistulae be closed in childhood, either surgically or by transcatheter coil occlusion. Liberthson et al. and Sunder et al. found that patients greater than 20 years of age had significantly higher rates of symptoms and complications of CAF [6,11]. But there is still discussion about recommending the elective closure of asymptomatic patients. A good argument in favour of a conservative approach is the unexpectedly high incidence of spontaneous closure of CAF [2]. On the one hand, the life long risk of the complications of the CAF is not known, but on the other hand any intervention to close such fistulae is associated with risk of morbidity and the benefit of such intervention is questionable.

If the decision is made for elective closure surgical ligation versus coil embolisation should be individualized. Factors that favor surgical ligation include large fistulas, multiple fistulous connections, extreme vessel tortuosity, presence of an aneurysm, need for concomitant distal bypass, or the presence of large branch vessels that can be inadvertently embolized [14]. Factors that favor transcatheter coil embolisation (TCE) include single drain site, older age, presence of a fistula arising as an accessory coronary artery and absence of an adjacent vessel [15].

Surgical ligation results in a high closure rate if there are not multiple sites, but the major disadvantage is the morbidity associated with the surgery. Some disadvantages associated with catheter closure of the fistulae are transient arrhythmias, coil embolization into the great vessels or recoil into the major coronary artery leading to acute mycardial infarction and occasionally sudden death. Also, complete obliteration of the CAF can be achieved in only 80–85% of the patients [16].

Differential diagnosis includes persistent ductus arteriosus, pulmonary arteriovenous fistula, ruptured sinus of Valsalva aneurysm, aortopulmonary window, prolapse of the right aortic cusp with a supracristal ventricular septal defect, internal mammary artery to pulmonary artery fistula and systemic arteriovenous fistula.

In conclusion, CAF are rare congenital lesions, especially our presented case. Doppler echocardiography is a proper noninvasive method to recognize CAFs and their courses. The management should be individualized according to the configuration of the CAF and the advantages and disadvantages of conservative therapy, coil embolization and surgical ligation.

**Figure 2:**
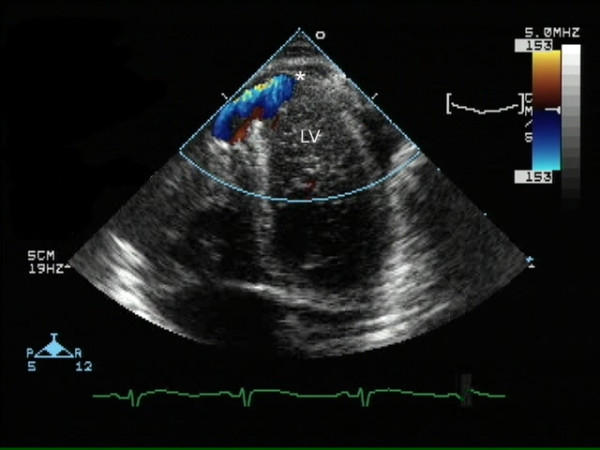
Apical site of the CAF insertion into the LV (*) and the adjacent muscular ventricular septal defect shown by color Doppler.
